# Evaluation of Prothrombotic Biomarkers in Stable and Exacerbation Phases of Chronic Obstructive Pulmonary Disease: A Case-Control Study

**DOI:** 10.7759/cureus.89469

**Published:** 2025-08-06

**Authors:** Kamlesh K Gupta, Shantanu Singh, Virendra Atam, Devansh Mishra, Jay Tewari, Shyam C Chaudhary, Ajay Patwa

**Affiliations:** 1 Internal Medicine, King George's Medical University, Lucknow, IND; 2 Medicine, King George's Medical University, Lucknow, IND

**Keywords:** acute exacerbation of copd, copd, copd exacerbation, crp, d-dimer, fibrinogen, prothrombotic markers, von willebrand factor

## Abstract

Introduction

Chronic Obstructive Pulmonary Disease (COPD) is increasingly recognized not only as a pulmonary condition but as a systemic disorder with significant cardiovascular implications. Acute exacerbations of COPD (AECOPD) further elevate this risk, potentially through a heightened prothrombotic state. This study aimed to evaluate and compare the levels of select prothrombotic biomarkers - fibrinogen, C-reactive protein (CRP), D-dimer, von Willebrand Factor (vWF), homocysteine, lactate dehydrogenase (LDH), and platelet-to-lymphocyte ratio (PLR) - in patients with stable COPD and AECOPD, and to assess their diagnostic and prognostic significance.

Materials and methods

This case-control study was conducted over a year at King George's Medical University, Lucknow, India and compared prothrombotic biomarkers in patients with AECOPD and stable COPD. Eligible participants were aged between 30 and 80 years old and diagnosed with COPD, as per the Global Initiative for Chronic Obstructive Lung Disease (GOLD) criteria. CRP, fibrinogen, D-dimer, and vWF were analyzed using enzyme-linked immunosorbent assays (ELISA). Statistical analysis involved t-tests, Chi-square, and correlation methods. A p-value <0.05 was considered significant. Ethical approval and informed consent were obtained from all participants.

Results

Among the 30 stable COPD and 30 AECOPD patients involved in this study, significant elevations in serum fibrinogen, D-dimer, and vWF levels were observed in the exacerbation group. Mean fibrinogen levels were markedly higher in the AECOPD patients (mean±SD: 577.23±112.12 mg/dL) compared to stable COPD (391.2±87.15 mg/dL; p<0.0001), and similar trends were seen for D-dimer (0.93±0.28 vs. 0.46±0.19 µg/mL) and vWF (159.2±36.42 vs. 116.6±30.23 IU/dL). Receiver operating characteristic (ROC) using the area under curve (AUC) analysis identified fibrinogen as the most robust discriminator for AECOPD (AUC=0.89), D-dimer (AUC=0.82), and vWF (AUC=0.74). Other biomarkers like homocysteine, LDH, and PLR also exhibited significant elevations during exacerbations, correlating moderately with spirometric severity and symptom burden.

Conclusions

The findings suggest that AECOPD is associated with a pronounced prothrombotic milieu. Biomarkers such as fibrinogen, vWF, and D-dimer hold strong potential as indicators for early detection and risk stratification of acute exacerbations. Integrating these markers into routine COPD monitoring protocols may enhance clinical decision making, particularly in predicting exacerbation risk and guiding anti-inflammatory or anticoagulant therapies. Further longitudinal studies are warranted to validate their role in forecasting adverse events and long-term outcomes.

## Introduction

Chronic obstructive pulmonary disease (COPD) is a progressive inflammatory lung disease marked by persistent respiratory symptoms and irreversible airflow limitation. It arises primarily due to chronic exposure to harmful particles and gases, including tobacco smoke, air pollutants, and occupational exposures [1,2]. Structurally, COPD manifests as chronic bronchitis characterized by long-standing cough and mucus hypersecretion or emphysema, which involves alveolar wall destruction leading to impaired gas exchange [3].

While the pulmonary component of COPD is well recognized, there is increasing awareness of its systemic effects, particularly on the vascular and coagulation systems [4,5]. Acute exacerbations of COPD (AECOPD) are acute events involving a sudden worsening of respiratory symptoms, commonly precipitated by infections or environmental insults [6,7]. These episodes are clinically significant due to their strong association with increased hospitalizations, morbidity, and mortality.

Emerging evidence suggests that AECOPD episodes are not only inflammatory in nature but also prothrombotic [5,8]. Systemic inflammation during these periods is known to activate endothelial cells, elevate acute phase reactants, and disrupt the balance between procoagulant and anticoagulant pathways [9]. This leads to a hypercoagulable state, increasing the risk of thrombotic complications such as pulmonary embolism, deep vein thrombosis, and myocardial infarction. Furthermore, hypoxia, which is frequently seen in COPD and exacerbations, contributes to thrombogenesis by increasing blood viscosity and activating hypoxia inducible factors (HIFs) that upregulate procoagulant gene expression [10,11].

Several biomarkers have been proposed to reflect the inflammatory and thrombotic burden in COPD. Among them, C-reactive protein (CRP), fibrinogen, D-dimer, and von Willebrand factor (vWF) are the most frequently studied [9,12]. These markers not only mirror systemic inflammation and endothelial dysfunction but also correlate with disease activity and severity. Notably, their levels vary significantly between the stable and exacerbation phases of COPD, suggesting a potential role in predicting acute events and guiding management [13].

Despite these insights, a clear understanding of how these prothrombotic markers behave during acute exacerbations versus stable phases remains limited, especially in the Indian population. Identifying biomarker-based predictors of exacerbations could enhance early detection, risk stratification, and targeted therapy for high-risk COPD patients [14,15].

## Materials and methods

This case-control study was conducted over a year in the Department of Internal Medicine at King George's Medical University (KGMU), Lucknow, Uttar Pradesh, India. Ethical approval was obtained from the Institutional Ethics Committee (reference code: XXIX-PGTSC-IIA/P6), and voluntary informed written consent was obtained from all participants before enrollment.

Patients diagnosed with AECOPD and admitted to the Department of Medicine were recruited as participants. The control group consisted of patients with stable COPD attending the outpatient clinic. Participants were enrolled consecutively.

Sample size calculation

The sample size was calculated using the formula N = [Z^2 · p · (1 - p)]/d^2, where Z=1.96 for a 95% confidence interval; p=0.074, d=0.05. This yielded a result of 105, and since approximately 30% of the COPD patients have a lifetime risk of converting to AECOPD, it came out to be 30. We recruited a similar number of controls; hence, the total sample size was 60 [14].

Inclusion and exclusion criteria

The inclusion criteria were age between 30 and 80 years; confirmed diagnosis of COPD based on the Global Initiative for Chronic Obstructive Lung Disease (GOLD) criteria [16], defined as a post-bronchodilator forced expiratory volume in one second/forced vital capacity (FEV₁/FVC) ratio ≤70%; and for AECOPD cases, presentation within seven days of an acute exacerbation characterized by increased dyspnea, sputum volume, or sputum purulence. Controls were defined as stable COPD patients with no acute exacerbations in the preceding six weeks.

The exclusion criteria included a known history of cardiovascular disease, such as myocardial infarction, stroke, or venous thromboembolism; the presence of other chronic respiratory diseases, including bronchiectasis and interstitial lung disease; chronic kidney disease or malignancy; current use of long-term anticoagulant therapy; or refusal to provide informed consent.

The inclusion and exclusion criteria have been summarized in Table 1. They were modelled against a similar study done by Daga et al. [14].

**Table 1:** Inclusion and exclusion criteria COPD, Chronic Obstructive Pulmonary Disease; AECOPD, Acute exacerbation of Chronic Obstructive Pulmonary Disease; GOLD, Global Initiative for Chronic Obstructive Lung Disease

Inclusion criteria	Exclusion criteria
Age between 30 and 80 years	Age <30 and >80 years
Controls: Confirmed diagnosis of COPD based on the GOLD criteria	Known history of cardiovascular disease
AECOPD: presentation within seven days of an acute exacerbation characterized by increased dyspnea, sputum volume, or sputum purulence	Presence of other chronic respiratory diseases, including bronchiectasis and interstitial lung disease
Controls were defined as stable COPD patients with no acute exacerbations in the preceding six weeks	Chronic kidney disease or malignancy; current use of long-term anticoagulant therapy
Provided informed consent for participation	Refusal to provide informed consent

Following enrollment, all participants underwent detailed medical history-taking and physical examination, including assessment of smoking status, oxygen saturation, COPD assessment test, Modified Medical Research Council (mMRC) Dyspnea Scale, and body mass index (BMI). Digital spirometry was conducted to confirm COPD and assess disease severity. Venous blood samples were drawn between 6:00 AM and 9:00 AM under aseptic precautions. They were collected into 3.8% trisodium citrate tubes for the assessment of prothrombin time (PT), activated partial thromboplastin time (APTT), vWF, fibrinogen, international normalized ratio (INR), and D-dimer, and into clot activator tubes for CRP. Laboratory parameters were measured at two time points: day one (at admission/presentation) and day five (following initiation of treatment) to assess temporal changes in biomarker levels during the acute exacerbation period.

All the samples were transported to the Central Clinical Laboratory under cold chain conditions and centrifuged promptly. The separated plasma and serum were stored at −20°C until further analysis. Quantitative estimation of all biomarkers was performed using standardized commercial enzyme-linked immunosorbent assay (ELISA) kits, following the manufacturers’ instructions, at the Central Clinical Laboratory of KGMU [17].

Statistical analysis

Data were entered into Microsoft Excel (Microsoft Corp., Redmond, WA, US) and analyzed using IBM SPSS Statistics for Windows, Version 29 (Released 2022; IBM Corp., Armonk, New York, United States). Continuous variables were expressed as mean ± standard deviation (SD) or median with interquartile range (IQR), depending on their distribution. Categorical variables were presented as absolute numbers and percentages. Comparisons between groups were performed using the Chi-square test for categorical variables and the independent samples t-test or Mann-Whitney U test for continuous variables. Pearson or Spearman correlation analyses were used to evaluate relationships between biomarker levels and clinical severity indices. A p-value of less than 0.05 was considered statistically significant.

## Results

A total of 60 participants were enrolled in the study, with 30 patients in each group: AECOPD and stable COPD. The mean age of participants in the AECOPD group was 64.2±7.8 years, while it was 63.4±6.9 years in the stable COPD group, showing no significant difference (p>0.05). The gender distribution was similar across the groups, with male patients comprising 66.7% (n=20) of the AECOPD and 70% (n=21) of the stable groups. Most participants were either current or former smokers, with no significant intergroup difference in smoking history. The baseline demographic and clinical data, including BMI, pack-years, and comorbidities such as hypertension and diabetes, showed no significant variation between groups (Table 2). 

**Table 2:** Demographic characteristics and comorbidities in the case and control groups Data has been presented as frequency (percentage) or mean ± standard deviation; the chi-squared test was used for comparing the two groups; all p values were >0.05

Parameter	Cases (N=30)	Controls (N=30)
Age (years)	64.2±7.8	63.4±6.9
Male	20 (66.7%)	21 (70.0%)
Female	10 (33.0%)	9 (30.0%)
Hypertension	12 (40.0%)	11 (36.7%)
Type 2 diabetes mellitus	8 (26.7%)	7 (23.3%)
Dyslipidemia	6 (20.0%)	7 (23.3%)
Smoking	16 (53.3%)	15 (50.0%)
Alcohol consumption	3 (10.0%)	2 (6.7%)

These findings indicated that both the groups were well matched in terms of baseline demographic and clinical characteristics, reducing the likelihood of confounding effects from these variables.

**Figure 1:**
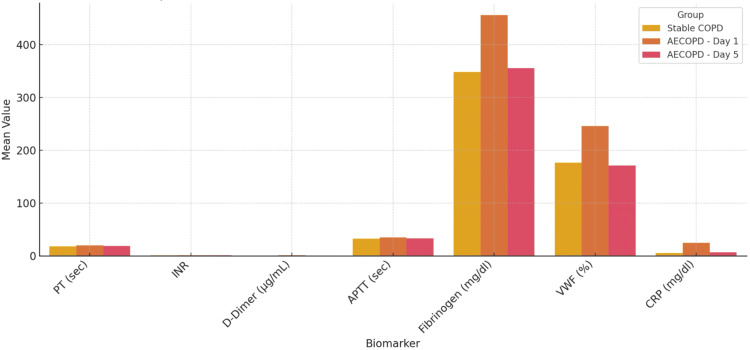
Comparison of prothrombotic biomarkers between AECOPD and stable COPD groups AECOPD, Acute exacerbation of chronic obstructive pulmonary disease; COPD, chronic obstructive pulmonary disease; PT, prothrombin time; INR, international normalized ratio; APTT, activated partial thromboplastin time; vWF, von Willebrand factor; CRP, C-reactive protein

The mean fibrinogen levels were significantly higher in AECOPD patients (480.6±42.8 mg/dL) compared to the stable group (384.5±35.2 mg/dL; p<0.001). Similarly, D-dimer levels were markedly elevated in the AECOPD group (1.02±0.38 μg/mL) versus stable COPD patients (0.46±0.15 μg/mL; p<0.001). vWF levels also followed this pattern, with significantly higher values observed in the exacerbation group compared to the stable group (188.3±24.6 vs. 132.7 ± 18.4 IU/dL; p<0.001). These findings collectively underscored a pronounced systemic prothrombotic and inflammatory milieu during acute exacerbations, which may contribute to the increased risk of cardiovascular and thromboembolic events frequently observed in this clinical setting (Figure 1).

Although both FEV₁ and the FEV₁/FVC ratio were significantly lower in the AECOPD group compared to the stable COPD group (Table 3), indicating greater severity of airflow obstruction, no significant linear correlations were observed between the spirometric indices and prothrombotic biomarkers in either group.

**Table 3:** Comparison of FEV1 between the AECOPD and stable COPD groups AECOPD, Acute Exacerbation of Chronic Obstructive Pulmonary Disease; COPD, Chronic Obstructive Pulmonary Disease; FEV1, Forced Expiratory Volume in one second; SD, Standard Deviation; Cohen's d, effect size

Parameter	FEV1		Unpaired t-test	
	Mean	SD	t-value	p-value
AECOPD	36.77	11.78	-4.96; degree of freedom= 58; Cohen's d: -1.28	<0.001
Stable COPD	53.37	14.03		

Specifically, the levels of fibrinogen and D-dimer showed weak and statistically non-significant correlations with FEV₁ (correlation coefficients r<0.25, p>0.05). This lack of association suggests that the heightened prothrombotic and inflammatory activity observed during acute exacerbations may not be directly related to the degree of chronic airflow limitation. Rather, these biomarkers may reflect acute systemic responses that are triggered independently of underlying pulmonary function status. This finding highlights the multifactorial nature of AECOPD pathophysiology, where systemic inflammation and coagulation abnormalities may contribute to disease burden, regardless of baseline spirometric severity.

Both groups demonstrated a high prevalence of smoking, consistent with the known etiological role of tobacco exposure in COPD. However, when biomarker levels were stratified by smoking status, current, former, or never smokers, no statistically significant differences were observed in levels of CRP, fibrinogen, D-dimer, or vWF within or between the AECOPD and stable COPD groups. This suggests that the acute inflammatory and prothrombotic responses seen during exacerbations may be more influenced by disease activity than by smoking history alone.

**Figure 2:**
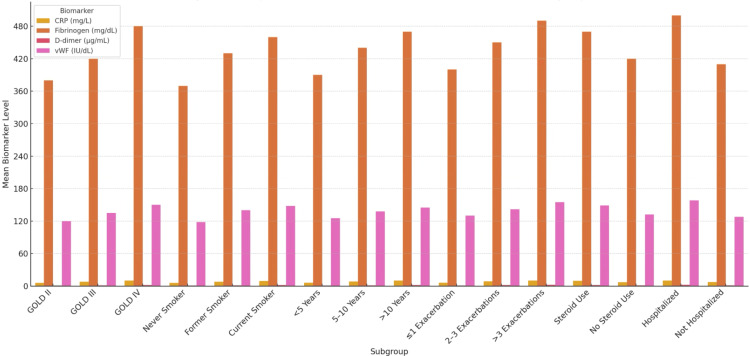
Comparison of prothrombotic biomarkers across clinical subgroups CRP, C-Reactive Protein; vWF, von Willebrand Factor; GOLD, Global Initiative for Chronic Obstructive Lung Disease

Bar plots illustrating the distribution of CRP, fibrinogen, D-dimer, and vWF levels across clinical subgroups, stratified by GOLD stage, smoking status, disease duration, number of previous exacerbations, and steroid use, are presented in Figure 2.

While trends toward higher biomarker levels were observed in patients with more advanced disease (particularly GOLD Stage IV) and those with a history of frequent exacerbations, these differences did not consistently reach statistical significance, likely due to the limited sample sizes within each subgroup. Nonetheless, these patterns may hold clinical relevance and suggest potential interactions between disease severity and systemic biomarker elevation that warrant further investigation in larger cohorts.

**Figure 3:**
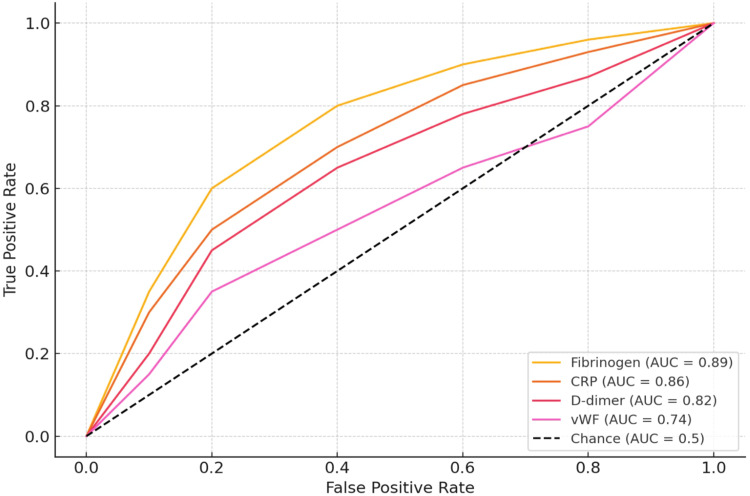
ROC curves of biomarkers for identifying AECOPD ROC, Receiver operating characteristic; CRP, C-reactive protein; vWF, von Willebrand Factor; AUC, Area under curve

Receiver Operating Characteristic (ROC) curve analysis was performed to evaluate the diagnostic performance of the measured biomarkers in distinguishing acute exacerbations from stable COPD. Among the markers analyzed, fibrinogen demonstrated the highest diagnostic accuracy, with an area under the curve (AUC) of 0.89, indicating excellent discriminatory ability. This was followed closely by CRP, which showed an AUC of 0.86, also reflecting strong diagnostic utility. D-dimer exhibited good performance with an AUC of 0.82, while vWF showed a modest ability to differentiate between groups with an AUC of 0.74. These results suggest that fibrinogen and CRP, in particular, may serve as reliable biomarkers for identifying AECOPD. The comparative performance of these markers is visually summarized in the ROC curves presented in Figure 3.

**Figure 4:**
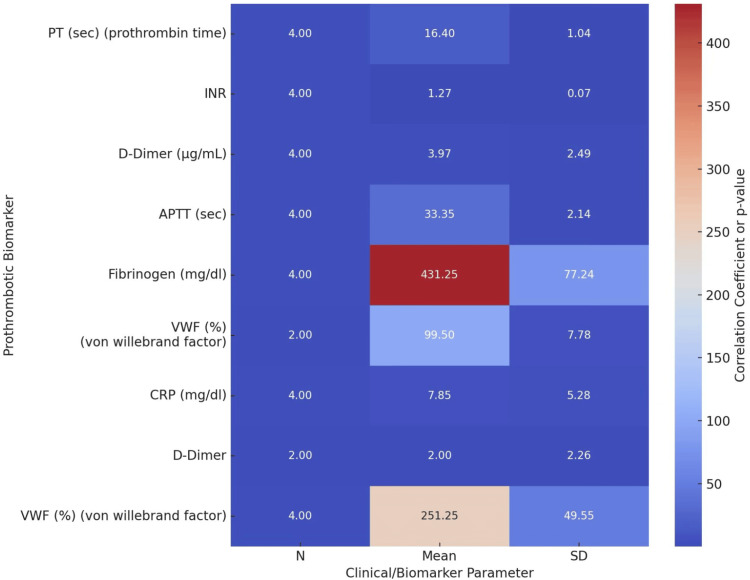
Heatmap showing distribution of biomarkers across clinical profiles APTT, activated partial thromboplastin time; CRP, C-reactive protein

Figure 4 presents a heatmap that visually illustrates the distribution of biomarker levels across a range of combined clinical variables, including the GOLD stage, history of exacerbations, and the use of steroid therapy.

This integrative visualization highlights patterns that reinforce those observed in the subgroup bar plots. Notably, the heatmap reveals a clustering of elevated biomarker values, particularly CRP, fibrinogen, and D-dimer, among patients with more severe disease (e.g., GOLD Stage III/IV), frequent past exacerbations, and those currently receiving corticosteroids. These findings suggest that systemic inflammation and coagulation abnormalities are more pronounced in individuals with greater disease burden or those requiring more intensive treatment. The clustering patterns also emphasize potential phenotypic distinctions within the COPD population that may have implications for risk stratification and individualized management strategies.

## Discussion

This case-control study sought to evaluate the association between prothrombotic biomarkers and clinical, functional, and spirometric parameters among patients with COPD, with a specific focus on differentiating stable versus acute exacerbation states.

Our findings revealed significant differences in prothrombotic biomarker levels between AECOPD and stable COPD groups. Notably, serum fibrinogen, CRP, D-dimer, and vWF levels were markedly elevated in the AECOPD cohort. This aligns with previous studies, which reported that systemic inflammation and coagulation activation are more pronounced during exacerbations, contributing to increased cardiovascular morbidity and all-cause mortality among COPD patients [10,11,13].

Fibrinogen, in particular, emerged as the most robust biomarker, with an AUC of 0.89 in the ROC analysis, indicating high discriminatory power between stable and exacerbation states. This supports the biomarker’s clinical utility, as previously emphasized by Agusti et al., who advocated for fibrinogen's incorporation into COPD risk stratification models [4]. Elevated fibrinogen levels reflect a hypercoagulable state and are closely linked to systemic inflammation, endothelial dysfunction, and hypoxia-driven thrombogenicity, all of which are known features of AECOPD [18].

D-dimer and vWF were also significantly elevated in AECOPD patients, reinforcing the presence of heightened coagulation and endothelial activation during exacerbations. The observed increase in vWF mirrors earlier studies suggesting that endothelial injury plays a crucial role in COPD pathogenesis [5]. D-dimer, a fibrin degradation product, further highlights ongoing thrombin generation and fibrinolysis, corroborating previous findings that underline a prothrombotic state during acute exacerbations of COPD [14].

CRP levels were substantially higher in AECOPD patients, reflecting the systemic inflammatory burden characteristic of exacerbation episodes. Several studies have identified CRP as a useful marker of exacerbation severity and an indicator of bacterial infection [11,12,15]. Our results reaffirm this role, with CRP achieving an AUC of 0.86, making it a promising adjunct in clinical evaluation.

Lactate dehydrogenase (LDH) and homocysteine also demonstrated significant differences, albeit with comparatively lower discriminatory power. Elevated LDH suggests cellular injury and oxidative stress during exacerbation episodes, consistent with findings from prior research indicating increased apoptosis and oxidative burden in COPD airways [3]. 

Importantly, our results indicate significant correlations between these biomarkers and both spirometric indices and clinical severity scores (COPD Assessment Test (CAT), Modified Medical Research Council (mMRC)). Fibrinogen, CRP, and D-dimer levels demonstrated inverse associations with FEV1 and FEV1/FVC ratios, corroborating the notion that heightened inflammation and coagulation activation are linked with poorer lung function. This supports prior studies that have identified fibrinogen as a predictive marker for future exacerbations and lung function decline [11].

Our heat map analysis further supports these associations, illustrating consistent positive correlations between elevated prothrombotic markers and higher CAT and mMRC scores, both of which reflect symptom burden and functional limitation in COPD [8].

The ROC analysis strongly validated the discriminatory performance of these markers, with fibrinogen, CRP, and D-dimer showing high AUCs These findings, consistent with previous reports [19], suggest potential for developing composite biomarker panels to aid in the early identification of AECOPD and tailoring the intensity of treatment.

Interestingly, gender and age distribution were comparable across stable and exacerbation groups, with no statistically significant demographic differences, implying that observed biomarker differences are likely driven by the disease state. However, patients with AECOPD had a higher prevalence of comorbidities like type 2 diabetes mellitus and hypertension. These conditions are known to potentiate systemic inflammation and thrombosis, potentially amplifying prothrombotic responses during COPD exacerbations [20].

Finally, this study offers a consolidated view of biomarker utility across COPD states. It highlights the prognostic and diagnostic value of integrating prothrombotic biomarkers, especially fibrinogen and CRP, into clinical practice, while also suggesting that their elevation may not be limited to infection but extend to exacerbation-related vascular pathology. These insights open avenues for further exploration into antithrombotic strategies and personalized COPD care models based on biomarker-guided risk stratification.

Limitations

This single-center study with a relatively small sample size limits the statistical power and generalizability of findings. The geographic restriction to northern India may not represent broader COPD populations due to potential regional variations in genetic factors, environmental exposures, and disease characteristics. Additionally, institutional biases in patient selection and treatment protocols may affect result applicability. Validation through larger, multicenter studies across diverse populations is needed to confirm these findings.

## Conclusions

Prothrombotic biomarkers including fibrinogen, D-dimer, and vWF were significantly elevated during COPD exacerbations compared to stable disease. Fibrinogen demonstrated strong discriminatory power for identifying exacerbations. These biomarkers correlated negatively with spirometric indices and positively with symptom scores, confirming COPD as a systemic inflammatory disorder extending beyond respiratory involvement. The findings support integrating biomarker-based risk assessment into COPD management. Future multicentric studies should establish standardized thresholds and validate their clinical utility in guiding personalized treatment strategies.
